# Effect of ablative fractional carbon dioxide laser combined with 1064 nm-Nd: YAG laser in the treatment of postburn hypertrophic scar

**DOI:** 10.12669/pjms.41.2.11088

**Published:** 2025-02

**Authors:** Lingqiao Li, Weichang Shen, Guozhong Lyu

**Affiliations:** 1 Lingqiao Li, Nanjing University of Traditional Chinese Medicine, Nanjing, Jiangsu Province, China, Department of Burn and Plastic Surgery, the Third Affiliated Hospital of Soochow University, Changzhou, China; 2 Weichang Shen, Department of Burn and Plastic Surgery, the Third Affiliated Hospital of Soochow University, Changzhou, China; 3 Guozhong Lyu Department of Burn and Plastic Surgery, the Affiliated Hospital of Jiangnan University, Wuxi, Jiangsu Province, China. Nanjing University of Traditional Chinese Medicine, Nanjing, Jiangsu Province, China

**Keywords:** 1064 nm-Nd: YAG laser, Ablative fractional carbon dioxide laser, Hypertrophic scars, Postburn

## Abstract

**Objective:**

To investigate the effect of ablative fractional carbon dioxide laser (AFCO2L) combined with 1064 nm-Nd: YAG laser in treating hypertrophic scars (HTS) in burn patients.

**Methods:**

This study retrospectively analyzed the medical records of 119 patients with postburn HTS admitted to The First People’s Hospital of Changzhou from March 2021 to October 2023. According to the treatment method, patients who received 1064 nm-Nd: YAG laser treatment were included in the YAG group, while patients who were treated by a combination of 1064 nm-Nd: YAG laser and AFCO2L comprised the combined group. Treatment effects, skin properties, and patients’ satisfaction with aesthetics were compared between the two groups.

**Results:**

The total efficacy of the combination group was significantly higher than that of the YAG group (*P*<0.05). After the treatment, skin elasticity, skin moisture, and pigmentation scores of the combined group were significantly higher than those of the YAG group (*P*<0.05). The level of satisfaction with the aesthetics was significantly higher in patients who received the combined treatment than those treated with YAG alone (*P*<0.05).

**Conclusions:**

A combination of AFCO2L and 1064 nm-Nd: YAG laser in the treatment of HTS is associated with a more significant effect, more significant improvement of skin properties, less pigmentation, and overall increased patient satisfaction with the aesthetics compared to the treatment with 1064 nm-Nd: YAG laser alone.

## INTRODUCTION

Burns, especially facial burns, have a profound impact on patients’ physical and mental health and facial aesthetics.^1^,^2^ Clinical studies have shown that the face is highly susceptible to thermal injury and irritation, leading to structural tissue damage and a higher risk of impaired melanogenesis, which can cause skin pigmentation and increase the visibility of hypertrophic scars (HTS).^3^,^4^ The degree of pigmentation directly correlates with the severity of the facial burn and the duration of exposure and may be further exacerbated by the long-term severe inflammation.^3^-^5^

Lasers, such as long pulse width 1064 nm-Nd: YAG laser and ablative fractional carbon dioxide laser (AFCO2L), are commonly used in patients with post-burn HTS and facial burn pigmentation.^6^,^7^ The 1,064 nm Q-switched Nd:YAG laser is a non-ablative and selective photo thermolysis system, and the AFCO2L is a minimally invasive procedure that uses a specialized laser to remove layers of scar tissue and promote the growth of new, healthier skin cells.^8^,^9^ The laser treatment is safe, allows to directly reach the dermis and stimulate collagen recombination,^6^-^9^ and, as demonstrated by current studies, is efficient in improving the skin condition of burn survivors.^7^,^10^

Several studies have reported the application of AFCO2L combined with 1064 nm-Nd: YAG laser in treating several skin conditions, such as atrophic facial acne scars, skin photo rejuvenation, and burn scars.^11^-^14^ However, the efficacy of the combined treatment in patients with post-burn HTS has not been widely confirmed in clinical practice. This retrospective retrospectively aimed to explore the effect of AFCO2L combined with 1064 nm-Nd: YAG laser on the treatment of post-burn HTS.

## METHODS

Clinical data of burn patients with HTS who received laser treatment in The First People’s Hospital of Changzhou from March 2021 to October 2023 were retrospectively selected. Patients were grouped according to the type of laser treatment received. Patients treated with 1064 nm-Nd: YAG laser comprised the YAG group, and patients treated with 1064 nm-Nd: YAG laser combined with AFCO2L comprised the combined group.

### Ethical Approval:

The hospital’s ethics committee approved this retrospective research: 2024-073, Date: 2024-04-03.

### Inclusion criteria:

- Patients diagnosed with post-burn HTS.
- Burn wound healing time ≥ 3 weeks.
- The course of scarring after wound healing within 1 year.
- Complete clinical data.
- Age ≥ 18 years old.
- Received 1064 nm-Nd: YAG laser, or 1064 nm-Nd: YAG laser combined with AFCO2L treatment.

### Exclusion criteria:

- Patients with other skin problems that may cause pigmentation (such as acne scars).
- Patients with facial skin diseases such as eczema.
- Patients with organic lesions in important organs.
- Unhealed wounds.
- Patients with keloids.
- Breastfeeding and pregnant women.

### Treatment:

Before treatment, patients were informed of the procedures of the treatment methods, possible adverse reactions, and postoperative precautions.

### YAG group (1064 nm-Nd: YAG laser):

Q-switched Nd: YAG (Spectra type) long pulse 1064 nm laser (Lutronic Corporation, Billerica, MA, USA) was used for the treatment. Before the procedure, the treatment site area of the patients was fully assessed and marked using a dermatoscope (CBS^®^WUHANBOSEELECTRONIC CO., LTD, Wuhan, China). Compound lidocaine cream (Ruyuan Dongyangguang Pharm, Shaoguan, Guangdong, China) was applied evenly to the treatment area, and the site was wrapped with cling film for 30 minutes and disinfected with 1% bromogeramine solution (Shandong Lircon Medical Technology Co., Ltd, Dezhou, Shandong, China). Laser output energy was adjusted to 5-10 J/cm^2^, and the spot was set to 3 mm. Low energy testing was performed, after which the output energy was adjusted based on erythema response. Treatment was carried out after clarifying the final output energy parameters. The procedure was performed once a month for a total of three treatments.

### Combined group (1064 nm-Nd: YAG laser combined with AFCO2L):

SmartXIDE type AFCO2L(DEKA M.E.L.A. SRL, Calenzano FI, Italy) was used. The trauma area was first treated with local anesthesia and disinfected. Then, 1064 nm-Nd: YAG laser treatment was implemented as described above. After the endpoint reaction at the trauma site, the treatment method was switched to AFCO2L. Parameters were as follows: spot size, 4 mm; output energy, 30-50 mJ; coverage rate, 3.7%; power, 25-30 W; spacing to 0.9 mm; focal length, 100 cm; and wavelength, 10600 nm. A combined procedure was carried out once a month, with three treatments.

After the treatment, both groups were given cold compress for 60 minutes and administered recombinant human epidermal growth factor spray 1-2 times daily.

### General information:

*Baseline data*, including gender, age, cause of injury, burn site, and scar area.

### Clinical effect after three months of treatment:

The effect was classified as follows^15^:

### Cured:

the disappearance of pigmentation and no difference in color with surrounding skin;

### Significantly effective:

improvement of pigmentation by ≥ 80%, with no difference in color compared to surrounding skin;

### Effective:

improvement of pigmentation between 60% to 79% with a certain difference with the surrounding skin;

### Invalid:

failure to meet the above standards. Cure, significant effectiveness, and effectiveness were included in the overall effective rate.

### Skin properties before and three months after the treatment:

Skin elasticity and moisture were evaluated using a skin detector. Multispectral irradiation was implemented using polarized light flash and standard white light to evaluate skin properties with a score range of 0-50 points. Higher scores indicated better skin elasticity and moisture status.

### Pigment deposition score before and three months after the treatment:

Skin pigment deposition was assessed using multispectral irradiation with polarized light flash and standard white light for automatic evaluation. The score range was 0-50 points, with a higher score indicating a better outcome.

### Satisfaction with the level of aesthetic:

A self-designed scale was used to assess the level of aesthetic, with a total of 10 points: very satisfied (9-10 points), satisfied (7-8 points), and dissatisfied (≤6 points); Treatment satisfaction= (very satisfied + satisfied)/total number of cases x 100%.

### Statistical Analysis:

SPSS22.0 version 17 was used for data analysis. For continuous variables, mean and standard deviation (SD) were calculated, and paired t-tests were used to determine the differences before and after the treatment within the same group. An independent sample t-test was used to compare the intergroups before and after the treatment. For categorical variables, frequency distribution was provided and expressed as a percentage. The Chi-square test was used to compare categorical variables, and the rank sum test was employed to compare level data. A *p*-value less than 0.05 was considered statistically significant. GraphPad Prism 8 software was used to draw graphs.

## RESULTS

This retrospective study included clinical data of 119 patients (70 males and 49 females), aged 18 to 58 years (mean age of 41.20 ± 10.50 years). There were 54 patients in the YAG group and 65 in the combined group, with no significant difference in the baseline data between the two groups (*P*>0.05) (Table-I). The overall effective rate of the combined group was significantly higher than that of the YAG group (*P*<0.05) (Table-II).

**Table-I:** Comparison of baseline data between two groups.

Baseline data	Combined group (n=65)	YAG group (n=54)	t/χ^2^	P
Gender				
Male	37 (56.92)	33 (61.11)	0.214	0.644
Female	28 (43.08)	21 (38.89)		
Age (year)	40.85±10.54	41.63±10.53	-0.404	0.687
Cause of injury				
Chemical burns	9 (13.85)	7 (12.96)	0.031	0.984
Flame burn	28 (43.08)	24 (44.44)		
High temperature burns	28 (43.08)	23 (42.59)		
Burned area				
Limb	26 (40.00)	22 (40.74)	0.027	0.999
Chest and abdomen	19 (29.23)	16 (29.63)		
Perioral region	16 (24.62)	13 (24.07)		
Others	4 (6.15)	3 (5.56)		
Trauma area (cm^2^)	35.39±10.06	37.01±9.54	0.895	0.373

**Table-II:** Comparison of treatment effects between two groups.

Group	n	Cured	Significantly effective	Effective	Invalid	Overall effective rate
Combined group	65	25 (38.46)	29 (44.62)	8 (12.31)	3 (4.61)	62 (95.39)
YAG group	54	12 (22.22)	18 (33.33)	15 (27.78)	9 (16.67)	45 (83.33)
*χ* ^2^						4.724
*P*						0.030

**Fig.1:**
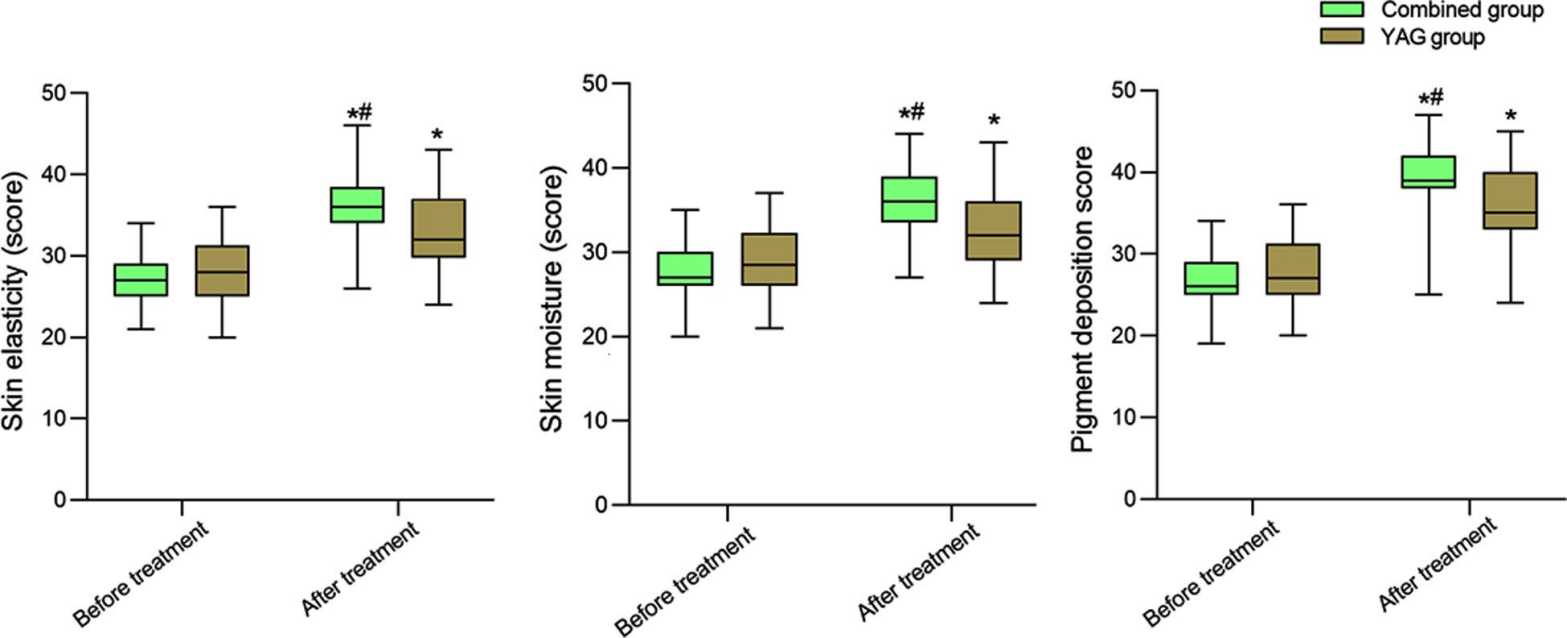
Comparison of skin elasticity, skin moisture, and pigmentation scores between two groups. Compare with the same group before treatment, ^*^P<0.05; Compared with the YAG group after treatment, ^#^*P*<0.05.

**Fig.2:**
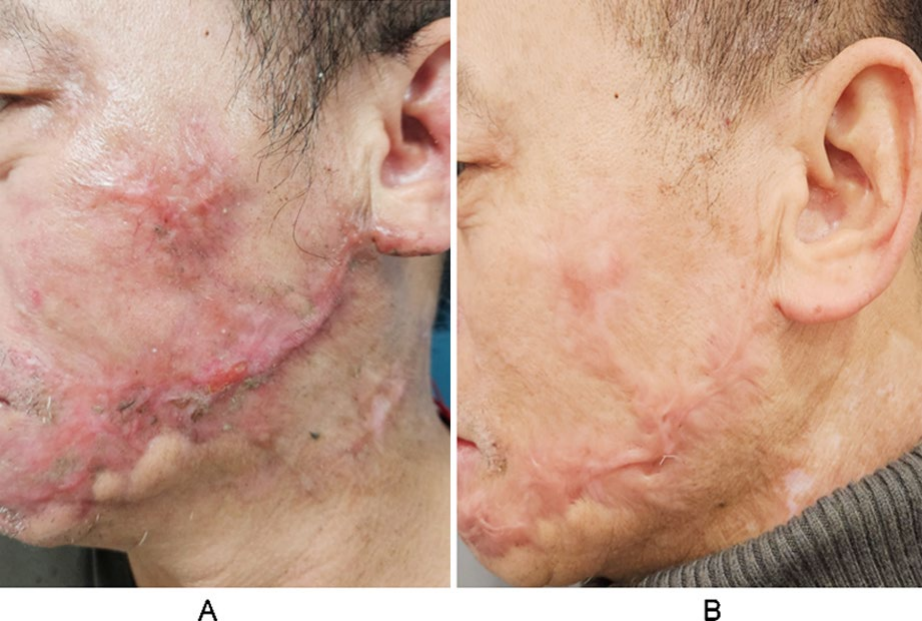
The postburn HTS before and after treatment. Male, 48 years old. A. Before treatment; B. After 3 months of treatment.

Before the treatment, skin elasticity, skin moisture, and pigmentation scores were comparable in the two groups (*P*>0.05). After the treatment, skin elasticity, skin moisture, and pigmentation scores of the two groups significantly increased compared to pre-treatment levels and were significantly higher in the combination group compared to the YAG group (*P*<0.05) (Fig-1). The satisfaction score of aesthetic appearance in the combined group (92.31%) was significantly higher than that in the YAG group (79.63%) (*P*<0.05) (Table-III) (Fig-2).

**Table-III:** Comparison of aesthetic satisfaction between two groups.

Group	n	Very satisfied	Satisfied	Dissatisfied	Overall satisfaction
Combined group	65	49 (75.38)	11 (16.92)	5 (7.69)	60 (92.31)
YAG group	54	25 (46.30)	18 (33.33)	11 (20.37)	43 (79.63)
*χ* ^2^					4.074
*P*					0.044

## DISCUSSION

This study showed that in patients with HTS, a combined AFCO2L and 1064 nm-Nd: YAG laser treatment had a significantly better therapeutic effect than 1064 nm-Nd: YAG laser treatment alone. The combined treatment was more effective in alleviating pigmentation and was associated with overall higher aesthetic satisfaction.

Numerous studies confirm that AFCO2L and 1064 nm-Nd: YAG lasers are commonly used in burn treatment.^10^,^16^-^19^ Lewis et al.^17^ found that AFCO2L treatment significantly changed the thickness and texture of burn scars. A study by Ruchiatan K et al.^18^ reported that a 1064 nm-Nd: YAG laser treatment of burn scars effectively destroyed melanosomes, making it a treatment choice for HTS and post-burn hyperpigmentation. Sipprell WH et al.^19^ also reported that 1064 nm-Nd: YAG laser treatment of burn scars significantly reduced the Vancouver Scar Scale (VSS) scores of the patients. Moreover, treatment was associated with significantly improved collagen structure and the number of blood vessels in each high-power field, good tolerance, and high safety. Other studies have found that 1064 nm-Nd: YAG laser treatment of burn scars can significantly alleviate scar viscoelastic deformation, skin roughness, and wrinkle depth.^20^,^21^ Consistent with these observations, this study further confirmed that 1064 nm-Nd: YAG laser had a high application value in post-burn pigmentation. However, the effective rate of the YAG group patients was 81.48%, and the satisfaction rate of aesthetic effect was 79.63%. These results indicate that although 1064 nm-Nd: YAG laser has a certain therapeutic value, there is still a gap between the overall effect and the expected clinical appearance after the treatment.

The present study found that in addition to improved skin elasticity, skin moisture, and pigmentation, the combined treatment also had better improvement effects than the YAG treatment alone. The overall efficacy of the combined treatment and satisfaction with aesthetic effects reached 95.39% and 92.31%, respectively, significantly higher than that of the YAG group. These results further confirmed that the treatment of post-burn HTS with combined 1064 nm-Nd: YAG laser and AFCO2L is feasible, can significantly improve treatment effect and skin condition, and is associated with higher satisfaction with the aesthetic effect. This is consistent with the previous research by Zheng Hulin et al.^21^. However, an RCT by Tawfic SO et al.^22^ that included 30 patients showed that the effect of AFCO2L combined with 1064 nm-Nd: YAG laser in the treatment of scar tissue did not increase significantly compared to using both methods on their own. We may speculate that this discrepancy may be related to the sample size or patient type.

The focus of different laser therapies varies.^18^-^20^,^23^ 1064 nm-Nd: YAG laser belongs to infrared light lasers, which are in the optimal fiber transmission range and have strong penetration. The produced thermal effect can heat and vaporize deeper melanin in the skin, thereby achieving the goal of melanin regression.^18^,^19^ AFCO2L can emit ultra-fine matrix laser beams, which can directly penetrate the dermis, promote gasification of abnormal proliferative tissues containing melanin, accelerate collagen synthesis, repair and rearrange collagen tissue, and thus, reconstruct the epidermis.^17^,^24^ This study confirmed that AFCO2L treatment can improve the treatment effect of post-burn HTS. However, we used a combination of AFCO2L and 1064 nm-Nd: YAG laser, which is different from the treatment protocols of previous studies.^25^-^27^ Chen HY et al.^25^ studied a combination of pulsed dye laser (PDL) and CO2 lattice laser for the treatment of HTS and showed that CO2 lattice laser combined with PDL treatment can timely inhibit vascular overgrowth, improve scar size, skin appearance, and pain and itching symptoms, and enhance clinical efficacy. A study by Huang Yue et al.^26^ demonstrated that compared to Er: YAG laser alone, the combination of Er: YAG laser and CO2 lattice laser has a more significant effect on treating facial acne scars. The above research results concluded that CO2 lattice laser combined with other laser treatment modalities can improve the treatment effect more effectively, with a higher degree of aesthetics.^25^,^26^

The current study confirmed the effectiveness and safety of AFCO2L combined with 1064 nm-Nd: YAG laser in treating post-burn HTS in terms of treatment effects, skin properties, and patients’ satisfaction with aesthetics. The results would allow to fill the research gap and provide a basis for clinical application and promotion of the combined treatment for patients with postburn HTS.

### Limitations:

This is a single-center retrospective analysis with a small sample size. Additionally, due to limited research time, no long-term follow-up investigation was conducted on the recurrence rate. Only a few observation indicators were analyzed in the current study, while other indicators, including pain intensity and complications after treatment, were not studied. Further high-quality studies with larger sample sizes, additional observation indicators, and longer follow-ups are needed to confirm these results.

## CONCLUSION

Combining AFCO2L and 1064 nm-Nd: YAG laser can improve skin properties, alleviate pigmentation, and enhance treatment effectiveness in treating HTS compared to 1064 nm-Nd: YAG laser alone. Combined treatment is associated with better patient satisfaction with the degree of aesthetics.

### Authors’ contributions:

**LL:** Concept, study design, literature each and manuscript writing.

**LL,WS** and **GL:** data collection, data analysis, interpretation and critical review.

**LL:** Revision of the manuscript and validation.

All authors have read, approved the final manuscript and are responsible for the integrity of the study.

## References

[1] Jeschke MG, Van Baar ME, Choudhry MA, Chung KK, Gibran NS, Logsetty S (2020). Burn injury. Nat Rev Dis Primers.

[2] Wan L, Zhou J, Li L (2022). Effects of punctate skin grafting combined with or without irrigation on skin graft survival, redness and swelling score and pain in treatment of large-area residual burn wounds. Pak J Med Sci.

[3] Guo XY, Xie WG (2021). Research progress on the mechanism of wound skin dys- pigmentation after burns. Zhonghua Shao Shang Za Zhi.

[4] Supp DM, Hahn JM, Lloyd CM, Combs KA, Swope VB, Abdel-Malek Z (2020). Light or Dark Pigmentation of Engineered Skin Substitutes Containing Melanocytes Protects Against Ultraviolet Light-Induced DNA Damage In Vivo. J Burn Care Res.

[5] Zhu Z, Sun Y, Zhou H, Tang HT (2020). [Advances in the research of clinical treatment of hyperpigmentation and hypopigmentation after burns]. Zhonghua Shao Shang Za Zhi.

[6] Kurup S, Travis TE, Shafy RAE, Shupp JW, Carney BC (2023). Treatment of burn hypertrophic scar with fractional ablative laser-assisted drug delivery can decrease levels of hyperpigmentation. Lasers Surg Med.

[7] Li L, Gao L, Zhao Y (2022). Effect of vitiligo treatment by compound Glycyrrhizin combined with fractional laser and Triamcinolone Acetonide injection on T Lymphocyte subpopulation. Pak J Med Sci.

[8] Kim YJ, Whang KU, Choi WB, Kim HJ, Hwang JY, Lee JH (2012). Efficacy and safety of 1,064 nm Q-switched Nd:YAG laser treatment for removing melanocytic nevi. Ann Dermatol.

[9] Betar N, Donovan M, Tyack Z, Warren J, McPhail SM, Vujcich E (2024). Recovery in patients undergoing ablative fractional carbon dioxide laser for adult hypertrophic burn scars:A longitudinal cohort study. Burns.

[10] Bunyaratavej S, Wanitphakdeedecha R, Ungaksornpairote C, Kobwanthanakun W, Chanyachailert P, Nokdhes YN (2020). Randomized controlled trial comparing long-pulsed 1064-Nm neodymium:Yttrium-aluminum-garnet laser alone, topical amorolfine nail lacquer alone, and a combination for nondermatophyte onychomycosis treatment. J Cosmet Dermatol.

[11] Asilian A, Salimi E, Faghihi G, Dehghani F, Tajmirriahi N, Hosseini SM (2011). Comparison of Q-Switched 1064-nm Nd:YAG laser and fractional CO2 laser efficacies on improvement of atrophic facial acne scar. J Res Med Sci.

[12] Tawfik AA, Hanafy NS, Ali RA (2024). Picosecond Nd:YAG versus Fractional CO2 Lasers in Management of Postburn Scars. Plast Reconstr Surg Glob Open.

[13] Bersy A, Abdel-Kawi F, Hamed Y (2021). Efficacy and safety of fractional CO2 laser versus long-pulsed Nd :YAG laser (1064&amp;#8201;nm) in skin photorejuvenation. Sci J Al-Azhar Med Faculty Girls.

[14] Cohen JL, Ross EV (2013). Combined fractional ablative and nonablative laser resurfacing treatment:a split-face comparative study. J Drugs Dermatol.

[15] Huang R, Wang H, Ba T, Yan Z, Zhou B, De Q (2021). Efficacy of ultra pulse CO2 lattice laser combined with long pulse width 1 064 nm Nd:YAG laser and platelet-rich plasma sequential therapy on burn scar. Chinese Journal of Injury Repair and Wound Healing (Electronic Edition).

[16] Altemir A, Boixeda P (2022). Laser Treatment of Burn Scars. Actas Dermosifiliogr.

[17] Lewis CJ, Douglas H, Martin L, Deng Z, Melton P, Fear MW (2023). Carbon dioxide laser treatment of burn-related scarring:Results of the ELIPSE (Early Laser Intervention Promotes Scar Evolution) prospective randomized controlled trial. J Plast Reconstr Aesthet Surg.

[18] Ruchiatan K, Suhada KU, Hindritiani R, Puspitosari D, Septrina R (2022). Combination of 1064 nm Long-Pulsed and Q-Switched Nd:YAG Laser for Facial Hypertrophic Scar and Hyperpigmentation Following Burn Injury. Int Med Case Rep J.

[19] Sipprell WH, Bell DE, Ibrahim SF (2020). Fractionally Ablative Er:YAG Laser Resurfacing for Thermal Burn Scars:a Split-Scar, Controlled, Prospective Cohort Study. Dermatol Surg.

[20] Khedr MM, Mahmoud WH, Sallam FA, Elmelegy N (2020). Comparison of Nd:YAG Laser and Combined Intense Pulsed Light and Radiofrequency in the Treatment of Hypertrophic Scars:A Prospective Clinico-Histopathological Study. Ann Plast Surg.

[21] Zheng H, Zheng J, Xie X (2022). Effect analysis of ultra pulse CO2 lattice laser combined with long pulse width 1064nm laser in the treatment of pigmentation in the posterior part of burns. Chin Med Cosme.

[22] Tawfic SO, El-Tawdy A, Shalaby S, Foad A, Shaker O, Sayed SS (2020). Evaluation of Fractional CO2 Versus Long Pulsed Nd:YAG Lasers in Treatment of Hypertrophic Scars and Keloids:A Randomized Clinical Trial. Lasers Surg Med.

[23] Madni TD, Lu K, Nakonezny PA, Imran JB, Cunningham HB, Clark AT (2019). Treating Hypertrophic Burn Scar With 2940-nm Er:YAG Laser Fractional Ablation Improves Scar Characteristics as Measured by Noninvasive Technology. J Burn Care Res.

[24] Yan H, Sun Y, Hu Y, Wu Y (2023). Ultrapulse carbon dioxide dot matrix laser for facial scar treatment:A meta-analysis. Int Wound J.

[25] Chen HY, Lei Y, OuYang HW, Gold MH, Tan J (2022). Experimental comparative study of the effect of fractional CO2 laser combined with pulsed dye laser versus each laser alone on the treatment of hypertrophic scar of rabbit ears. J Cosmet Dermatol.

[26] Huang Y, Zhang Q, Zhao X, Yuan J, Li G, Tu X (2021). The efficacy of exfoliative CO2 lattice laser combined with Er:YAG laser in the treatment of facial depressed acne scars. Chinese J of Laser Med.

[27] Kivi MK, Jafarzadeh A, Hosseini-Baharanchi FS, Salehi S, Goodarzi A (2024). The efficacy, satisfaction, and safety of carbon dioxide (CO2) fractional laser in combination with pulsed dye laser (PDL) versus each one alone in the treatment of hypertrophic burn scars:a single-blinded randomized controlled trial. Lasers Med Sci.

